# Photoelectrochemical and Raman characterization of In_2_O_3_ mesoporous films sensitized by CdS nanoparticles

**DOI:** 10.3762/bjnano.4.27

**Published:** 2013-04-11

**Authors:** Mikalai V Malashchonak, Sergey K Poznyak, Eugene A Streltsov, Anatoly I Kulak, Olga V Korolik, Alexander V Mazanik

**Affiliations:** 1 Belarusian State University, Nezalezhnastsi Av. 4, Minsk 220030, Belarus; 2 Institute of General and Inorganic Chemistry, National Academy of Sciences of Belarus, Surganova str., 9/1, Minsk 220072, Belarus

**Keywords:** cadmium sulfide (CdS), indium oxide (In_2_O_3_), nanoparticles, successive ionic layer adsorption and reaction (SILAR)

## Abstract

The method of successive ion layer adsorption and reaction was applied for the deposition of CdS nanoparticles onto a mesoporous In_2_O_3_ substrate. The filling of the nanopores in In_2_O_3_ films with CdS particles mainly occurs during the first 30 cycles of the SILAR deposition. The surface modification of In_2_O_3_ with CdS nanoparticles leads to the spectral sensitization of photoelectrochemical processes that manifests itself in a red shift of the long-wavelength edge in the photocurrent spectrum by 100–150 nm. Quantum-confinement effects lead to an increase of the bandgap from 2.49 to 2.68 eV when decreasing the number of SILAR cycles from 30 to 10. The spectral shift and the widening of the Raman line belonging to CdS evidences the lattice stress on the CdS/In_2_O_3_ interfaces and confirms the formation of a close contact between the nanoparticles.

## Introduction

In third-generation solar cells molecular dyes (in the Grätzel cells [1]) as well as nanoparticles of semiconducting metal chalcogenides (CdS, CdSe, CdTe, PbS, PbSe, Ag_2_S, Bi_2_S_3_ and others) immobilized onto the surface of mesoporous films of wide-bandgap oxides (TiO_2_, ZnO, SnO_2_, Nb_2_O_5_, Ta_2_O_5_, WO_3_, In_2_O_3_) are used as the sensitizing components [2–5]. It is considered that the best technique for the in situ deposition of such nanoparticles is the successive ionic layer adsorption and reaction (SILAR) method [6–11], which allows the precise control of the growth of the nanoparticles. To efficiently inject electrons from the photo-excited semiconductor nanoparticles or molecular dyes into the oxide matrix, the energy of the oxide conduction-band edge (*E*_c_) should be lower than the lowest unoccupied level (LUMO) of the sensitizer, to which the electrons from the highest occupied level are transferred under photoexcitation. The sensitizing efficiency of the photoelectrochemical system depends on (a) the efficiency of separation of the photo-induced charge-carriers at the sensitizer, (b) a wide-bandgap oxide interface that is mainly determined by the difference *E*_LUMO_ − *E*_c_, as well as (c) on the further transport of the charge carriers through the mesoporous oxide matrix to the substrate. Previous investigations have demonstrated that, in comparison to TiO_2_, In_2_O_3_ possesses higher electron affinity [12] allowing the use of sensitizers with a smaller bandgap *E*_g_ = *E*_LUMO_ – *E*_HOMO_. Besides, indium oxide is characterized by an essentially higher (by an order of magnitude) lifetime of the injected photoelectrons [12]. Interest in a more profound investigation of nanostructured In_2_O_3_ sensitization is also explained by the fact that the *E*_c_ level of In_2_O_3_ can be managed by the thermal treatment during preparation by the sol–gel method.

The aim of this work was to investigate the photoelectrochemical behavior of mesoporous nanocrystalline In_2_O_3_ films sensitized by CdS nanoparticles and to characterize these systems by using different methods (SEM, TEM, AES, UV–vis absorption, BET surface area measurements and Raman spectroscopy).

## Experimental

Mesoporous In_2_O_3_ films were prepared by spin-coating deposition of a colloidal indium hydroxide solution onto transparent ITO-coated glass or quartz (for optical measurements) substrates with a subsequent thermal treatment for 1 h in air. To study the effect of the spectral sensitization we used two kinds of In_2_O_3_ films: films annealed at 200 °C (hereafter referred to as In_2_O_3_(200)) and films annealed at 400 °C (hereafter referred to as In_2_O_3_(400)). The indium hydroxide sol was obtained as described in detail in [13]. Briefly, the indium hydroxide was precipitated by the titration of a 0.25 М In(NO_3_)_3_ aqueous solution with a concentrated NH_3_ solution under vigorous stirring at 0 °C. The precipitate was then thoroughly washed and ultrasonically treated after the addition of a small amount of nitric acid as a stabilizer to obtain a stable sol at a concentration of 120 g/L. To prepare indium oxide In_2_O_3_(400) films with mesoporous structure, block-copolymer Pluronic F127 (100 g/L) as a template material was added into the obtained indium hydroxide sol.

CdS nanoparticles were deposited onto the In_2_O_3_ films by the successive ion layer adsorption and reaction (SILAR) technique. This involved the dipping of the In_2_O_3_ film in a 1 M Cd(NO_3_)_2_ ethanolic solution for 5 min, rinsing it with distilled water, then dipping it for another 5 min in a 1 M Na_2_S aqueous solution and rinsing it again with distilled water. The deposition cycle was repeated 10–50 times, and finally, the sample was thoroughly rinsed with distilled water and air dried.

The prepared samples were characterized by SEM, TEM, BET surface area measurements, AES, photoelectrochemical methods, and UV–vis and micro-Raman spectroscopy. SEM and TEM images were obtained using a Hitachi S 4800 field emission scanning electron microscope and a LEO-906E transmission electron microscope, respectively. BET surface area measurements were carried out by using an ASAP2020MP analyzer of surface area and porosity. The Auger spectroscopy method in combination with ion etching was realized by using a PHI-660 Auger microprobe. Photoelectrochemical measurements were carried out in a standard two-compartment three-electrode cell involving a platinum counter-electrode and a Ag|AgCl|KCl (sat.) electrode as the reference electrode (+0.201 V versus SHE). The cell was controlled by a conventional programmable potentiostat. Photocurrent spectra were obtained by using a setup equipped with a high-intensity grating monochromator, a 1 kW xenon lamp and a slowly rotating light chopper (0.3 Hz). Spectral dependencies of the photocurrent were corrected for the spectral intensity distribution of the monochromator output. UV–vis absorption spectra of the films deposited onto quartz substrates were recorded on a Shimadzu UV-2550 spectrophotometer. Raman spectra were taken at room temperature by using a Nanofinder High End (Lotis TII, Belarus–Japan) confocal-microscope-based setup. Raman scattering was excited by using a 473 nm solid-state laser. The laser power level at the sample was maintained at ca. 25 μW to minimize laser-induced damage to the CdS nanoparticles. The backscattered light, not analyzed for its polarization, was dispersed by using a 0.55 m single grating spectrometer (spectral resolution better than 1 cm^−1^) and detected by a cooled CCD camera. The signal accumulation time was typically equal to 600 s. The spectral calibration with an accuracy better than 1 cm^−1^ was carried out by using a built-in gas-discharge lamp.

## Results and Discussion

**Figure 1:**
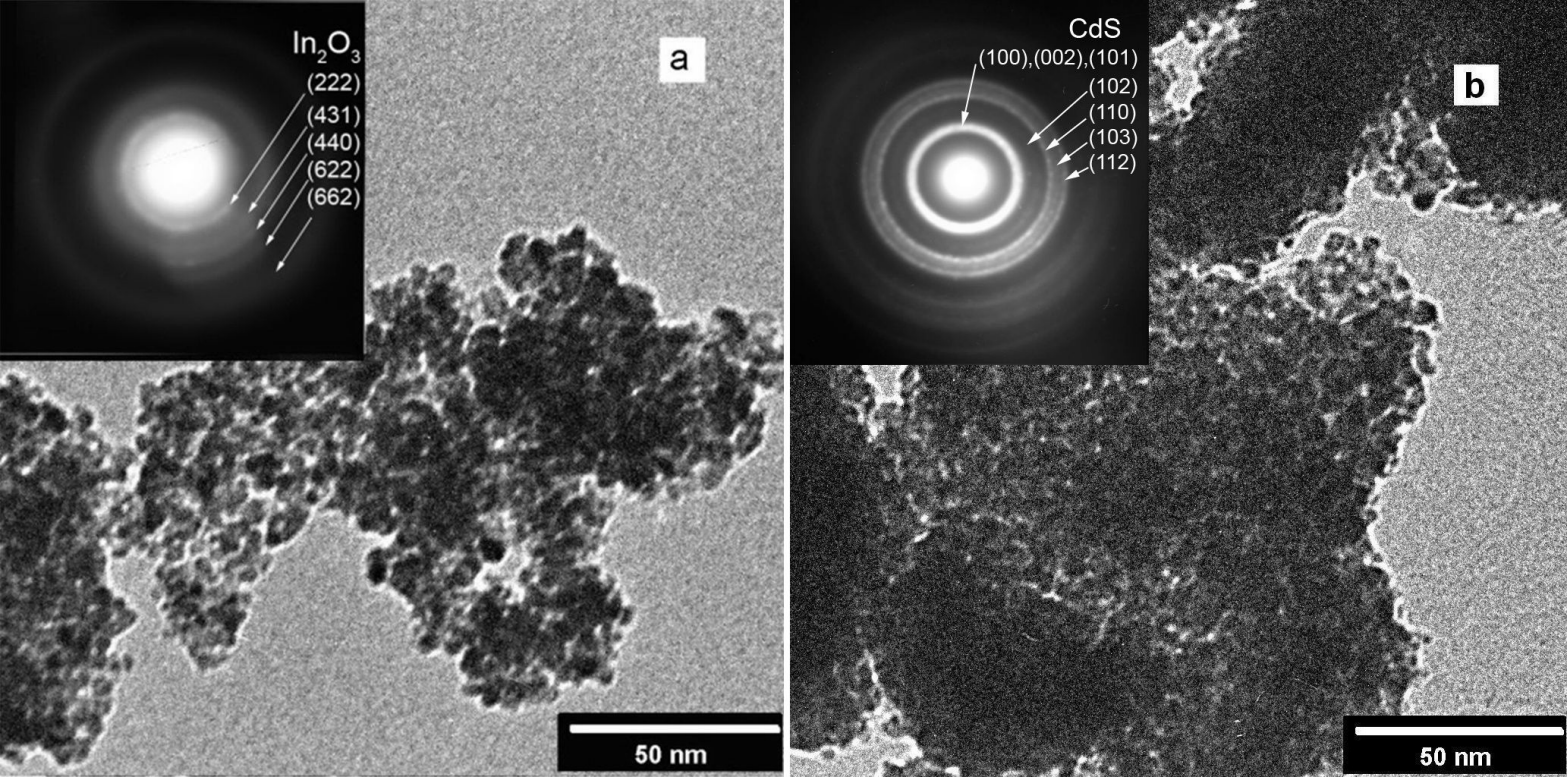
TEM images and electron diffraction patterns (insets) for In_2_O_3_(400) (a) and In_2_O_3_(400) after 30 SILAR CdS deposition cycles (b).

**Figure 2:**
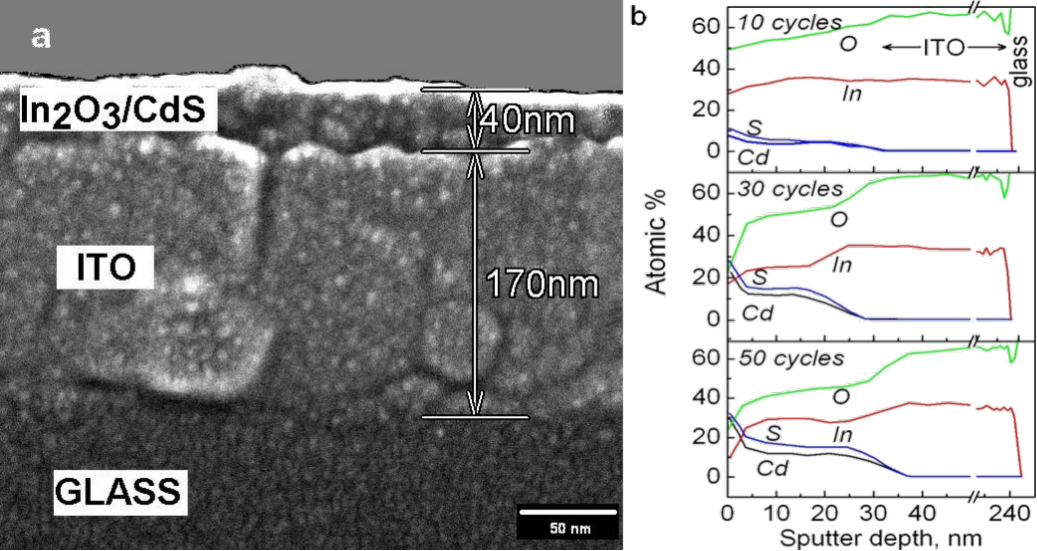
Cross-sectional SEM image of the In_2_O_3_(400) film after 30 SILAR CdS deposition cycles (a) and AES profiles for In_2_O_3_(400)/CdS structures with different numbers of SILAR cycles (b). Scaling from sputter time to depth in AES profiles was done on the basis of cross-sectional SEM data.

**Figure 3:**
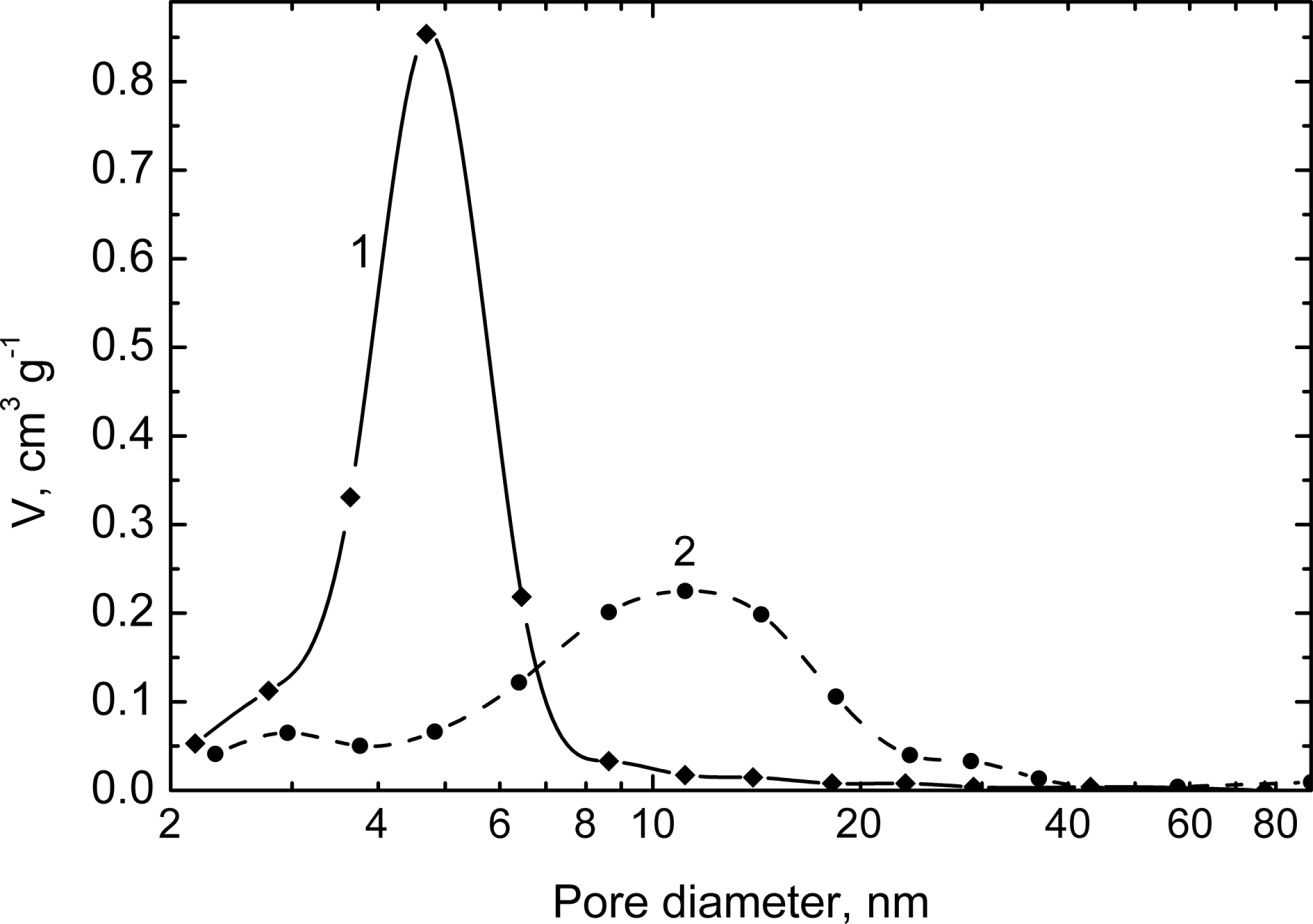
Barrett–Joyner–Halenda desorption-model pore-volume distribution of the In_2_O_3_(400) mesoporous films prepared with (1) and without (2) Pluronic F127.

TEM images of the prepared films demonstrating their highly porous and nanocrystalline structure are shown in Figure 1. The microdiffraction pattern (inset of Figure 1b) indicates the presence of hexagonal CdS in the sensitized In_2_O_3_ films. The cross-sectional SEM image of a film deposited on an ITO substrate shows that the film thickness is about 40 nm (Figure 2a). According to the BET data, the surface areas of the In_2_O_3_(400) films prepared with and without Pluronic F127 are 119 and 53 m^2^/g, respectively. It should be noted that the addition of Pluronic to the indium hydroxide sol leads to a significantly narrower pore-size distribution with an average pore diameter of ca. 5 nm (Figure 3).

**Figure 4:**
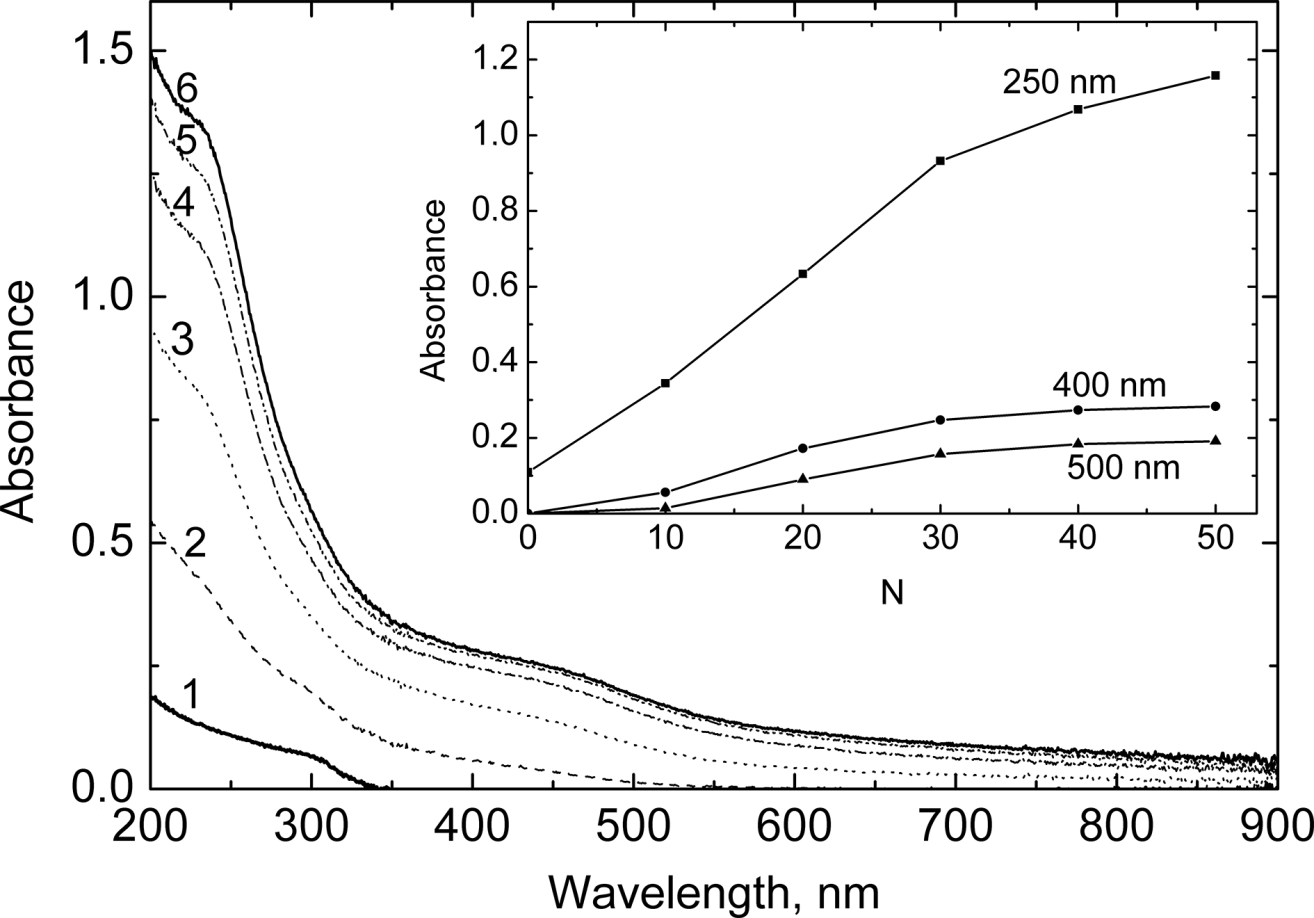
Absorption spectra of the In_2_O_3_(400) and In_2_O_3_(400)/CdS films deposited onto a quartz substrate. The number of SILAR cycles of the CdS deposition are as follows: 0 (1); 10 (2); 20 (3); 30 (4); 40 (5); 50 (6). The inset shows the dependencies of the absorbance at different wavelengths on the number of SILAR cycles.

Figure 4 shows the absorption spectra of the In_2_O_3_ films with deposited CdS nanoparticles. As can be seen clearly from Figure 4, the steepest rise in the absorbance is observed when increasing the number of SILAR cycles from 10 to 30. The absorbance does not change notably with the further deposition of CdS, indicating that the filling of the nanopore volume in In_2_O_3_ films with CdS particles occurs mainly during the first 30 cycles of SILAR deposition. According to the Auger profiles shown in Figure 2b, the film pores are filled rather uniformly at the initial stages of the SILAR deposition. Onwards, when the pore space is mostly filled, CdS nanocrystals continue to grow predominately on the top of the film.

**Figure 5:**
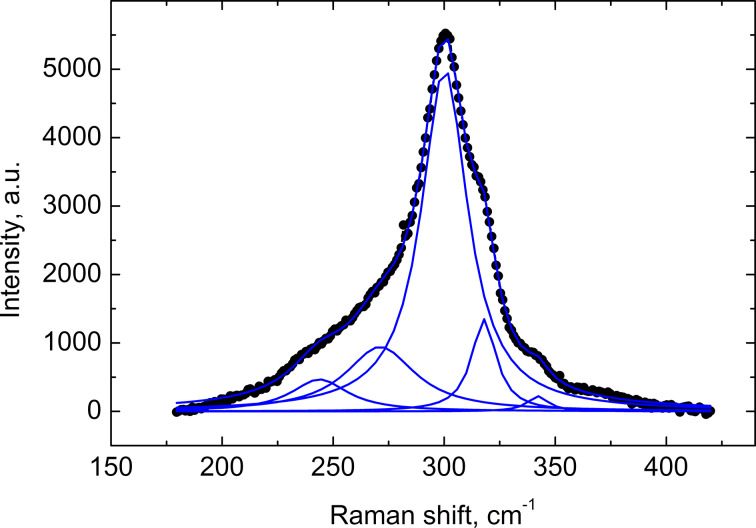
Raman spectrum of the In_2_O_3_(400)/CdS system prepared by using 40 SILAR cycles of CdS deposition after background subtraction (circles) and its fitting by the superposition of five Lorentz lines.

**Figure 6:**
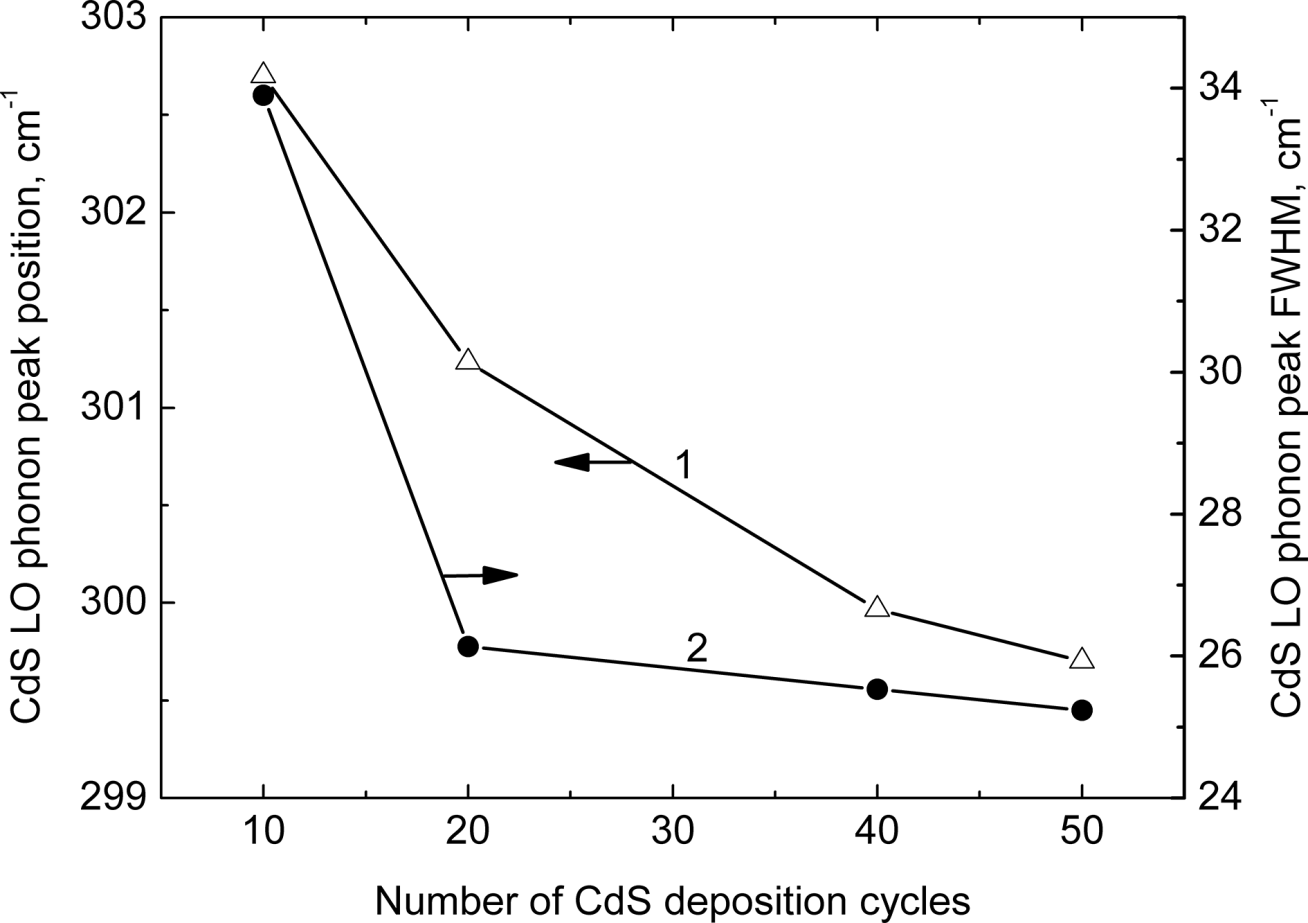
Dependence of the CdS LO phonon peak position (1) and FWHM (2) on the number of SILAR cycles.

The In_2_O_3_(400)/CdS films were also characterized by Raman spectroscopy. Raman spectra of all samples in the spectral range studied (0–1000 cm^−1^) show the CdS LO phonon mode (≈300 cm^−1^) with its two overtones, which correspond to two- (≈600 cm^−1^) and three-phonon (≈900 cm^−1^) processes. Figure 5 presents the Raman spectrum of the In_2_O_3_(400)/CdS system prepared by using 40 SILAR cycles. One phonon peak for CdS has a complex structure. The peak fitting by superposition of Lorentz lines allowed for the determination of position, full width at half maximum (FWHM) and relative intensity of the different components. The peak at ≈270 cm^−1^ can be associated with CdS surface phonons [14]. The peaks at ≈242 cm^−1^, ≈318 cm^−1^ and ≈342 cm^−1^ are not characteristic of In_2_O_3_ (which has Raman modes at approximately 308, 365, 504 and 637 cm^−1^ [15–16]) and are not observed for the In_2_O_3_ substrate without deposited CdS. The presence of these peaks in the Raman spectra can testify to the fact that the samples studied contain not only In_2_O_3_ and CdS, but also some additional phases formed during the SILAR process. High values of the FWHM of the LO phonon peak can point to both a significant degree of CdS nanocrystal disorder and size heterogeneity (Figure 6).

As can be seen from Figure 6, the CdS phonon energy decreases with increasing the number of SILAR cycles (i.e., the nanocrystal size). This effect can be explained in the following way: according to wavevector-relaxation [17] and continuum [18–19] models, the decrease of the size of the nanoparticles leads to a monotonic red shift of the phonon energy relative to the value characteristic of a bulk crystal. This effect was observed by a number of researchers who studied nanocrystals produced by colloidal chemistry methods [20–22]. However, in our experiments where the nanoparticles were grown in situ (directly on the indium oxide substrate) it is also necessary to take into account their interaction with the host material. A similar approach was applied in the works [23] and [24] to explain Raman spectra of CdS*_x_*Se_1−_*_x_* and CdSe embedded in different glass matrixes. The lattice mismatch between CdS and the host material can cause compressive stress in CdS nanocrystals which leads to an increase of the atomic interaction and hence to a phonon energy increase. The latter could explain the blue shift of the peak position that is observed when decreasing the number of SILAR cycles. In contrary, when the number of deposition cycles increases, the influence of the substrate diminishes and the CdS phonon energy decreases.

**Figure 7:**
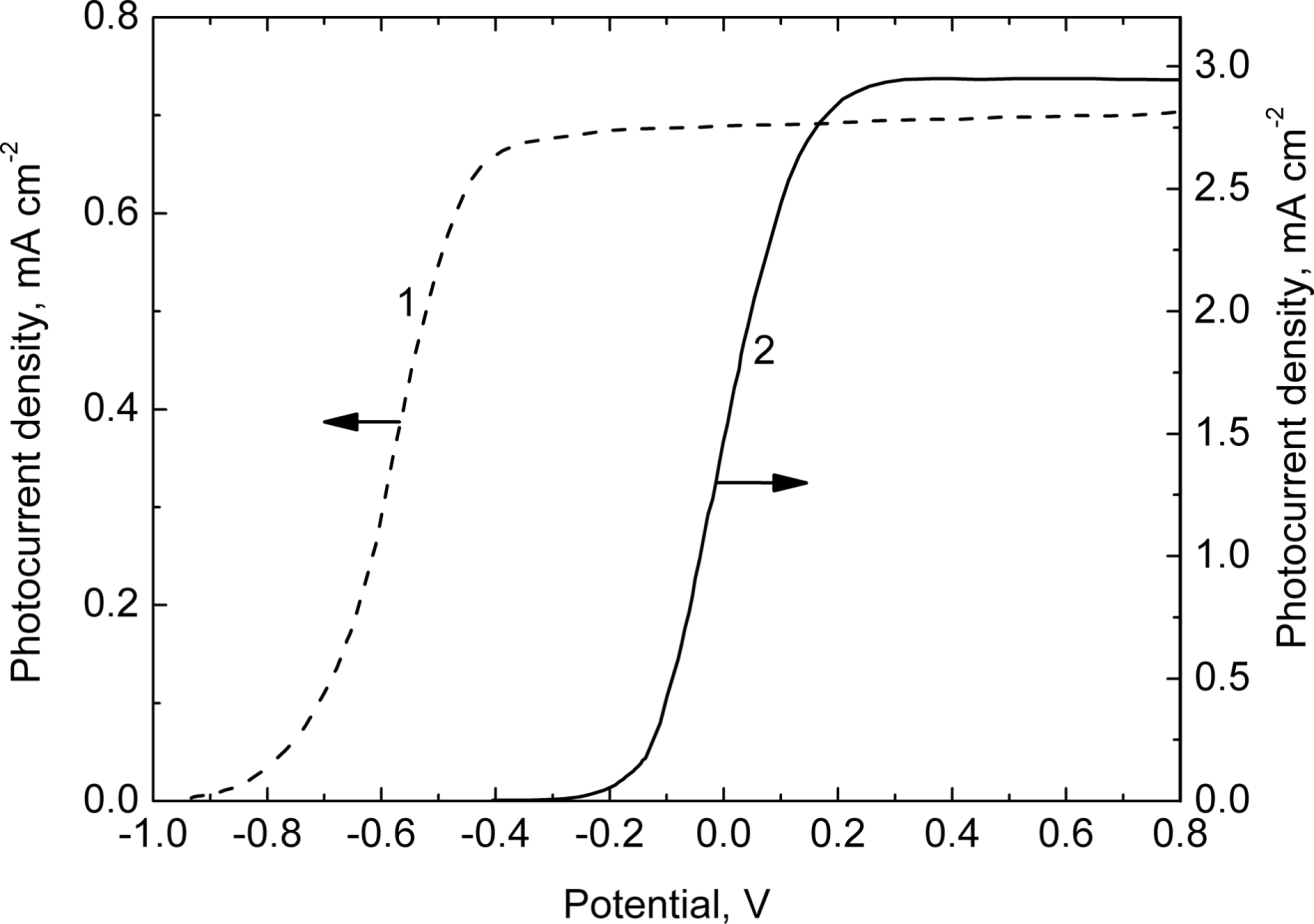
Photocurrent versus electrode-potential curves recorded under UV illumination of the mesoporous In_2_O_3_ electrodes annealed at 200 °C (1) and 400 °C (2). Electrolyte: 0.1 M KOH solution.

The In_2_O_3_ prepared films absorb predominantly UV light with λ < 400 nm since they have a relatively wide bandgap (2.87 eV for In_2_O_3_(200) and 2.73 eV for In_2_O_3_(400), indirect optical transitions) [13]. Under external polarization and UV illumination the In_2_O_3_ electrodes generate an anodic photocurrent because indium oxide is an n-type semiconductor. It is remarkable that the onset potential of the photocurrent is shifted to the positive direction by approximately 700 mV when increasing the temperature of the oxide annealing (Figure 7).

**Figure 8:**
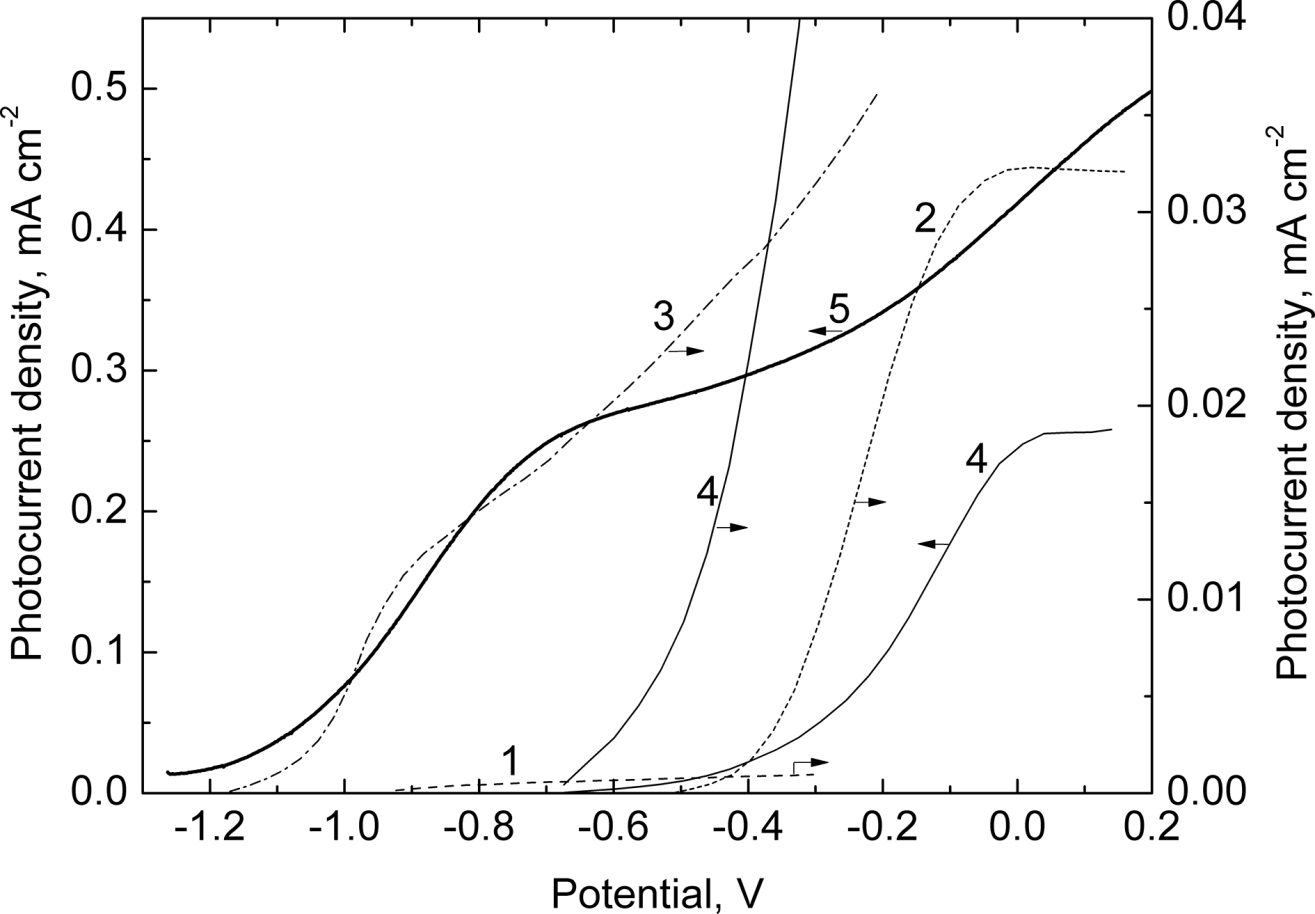
Photocurrent versus electrode potential curves recorded under visible-light illumination of the In_2_O_3_ (1, 2) and In_2_O_3_/CdS electrodes (3–5). The In_2_O_3_ films were annealed at 200 (1, 3) and 400 °C (2, 4, 5). The number of the SILAR cycles of CdS deposition was 10 (3, 4) and 50 (without the step of intermediate rinsing with distilled water, 5). Electrolyte: 0.1 М Na_2_S + 0.1 M Na_2_SO_3_ + 0.1 M NaOH solution.

Figure 8 shows the photocurrent–potential curves recorded under visible-light illumination of In_2_O_3_(200)/CdS and In_2_O_3_(400)/CdS heterostructures (curves 3 and 4) in an S^2−^-containing electrolyte. For comparison, similar dependencies for nonsensitized In_2_O_3_(200) and In_2_O_3_(400) electrodes are also presented (curves 1 and 2). The sensitized photocurrent for In_2_O_3_(400)/CdS is ten times higher than that for In_2_O_3_(200)/CdS. The onset potentials, *E*_on_, of the sensitized photocurrent correlate essentially with the corresponding *E*_on_ values for nonsensitized In_2_O_3_(200) and In_2_O_3_(400) and differ one from another by approximately 500 mV.

It is known that the *E*_on_ value as a first approximation can be identified as the energy level of the conduction-band edge (*E*_c_) of a semiconductor. The thinnest (approximately 1 nm) hydroxide layer has been previously shown [13] to be formed at the In_2_O_3_(200) surface. This layer seems to be of crucial importance in changing the potential drop in the Helmholtz layer of the electrode and consequently in changing the *E*_c_ level on the potential scale. Thus, it can be concluded that the energy diagram of the In_2_O_3_/CdS heterostructure is primarily determined by the structure of the oxide surface being in contact with the electrolyte. By controlling the state of this surface it is possible to change the *E*_on_ value and therefore the photovoltage of the corresponding PEC cell.

Very fine CdS particles (approximately several nanometers) seem to form on the surface of mesoporous In_2_O_3_ at a relatively small number of SILAR cycles (up to 30–40), as evidenced by the revealed quantum-size effect. The further increase in the SILAR cycle number (up to 50 and above) can result in the formation of a bulk CdS film on the In_2_O_3_ surface. To simulate the transition from nanoparticles to the bulk cadmium sulfide, a thick (several hundreds of nanometers) CdS film was deposited onto the surface of In_2_O_3_(400). When preparing this film, the step of intermediate rinsing of the samples with distilled water necessary to remove surplus Cd^2+^ and S^2−^ ions from the surface was omitted.

When depositing the bulk CdS, the isotype n-In_2_O_3_/n-CdS heterojunction with the external narrow-bandgap semiconductor is formed. In contrast to nanoparticles, the space-charge region can be formed in this bulk semiconductor. As can be inferred from the photoelectrochemical behaviour of this system (Figure 8, curve 5), its solid-state heterojunction is not a barrier for photogenerated electrons. A distinctive feature of the photocurrent–potential curves of the heterojunction is the sharp shift of the onset potential to a more negative potential (*E*_on_ = −1.2 V). In this case, the *E*_on_ value is already determined by the position of the conduction band edge of CdS.

**Figure 9:**
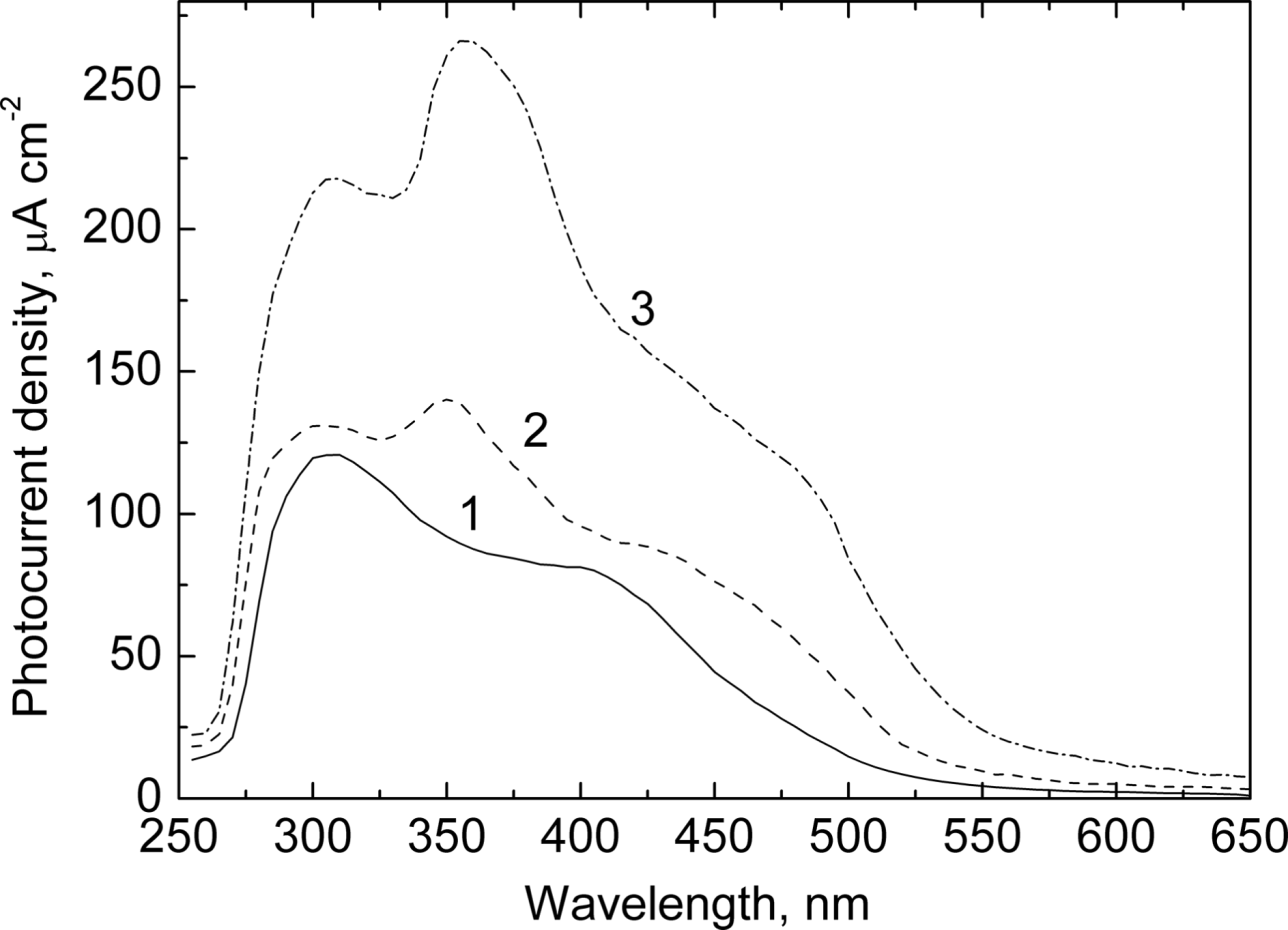
Photocurrent spectra of In_2_O_3_(400)/CdS film electrodes in 0.1 М Na_2_S + 0.1 M Na_2_SO_3_ + 0.1 M NaOH solution. The spectra were measured at a potential of −0.1 V versus Ag/AgCl. The number of the SILAR cycles of CdS deposition was 10 (1), 30 (2) and 50 (3).

Figure 9 presents the photocurrent spectra of the In_2_O_3_(400)/CdS heterostructure. The SILAR deposition of CdS nanoparticles onto the In_2_O_3_ films leads to an extension of the long-wave edge of the photocurrent spectra up to 550 nm. Although the photocurrent increases in the whole spectral region studied when increasing the number of SILAR cycles, the greatest increase in photocurrent is observed at λ > 450 nm, i.e., outside the region of the indium oxide bandgap absorption.

**Figure 10:**
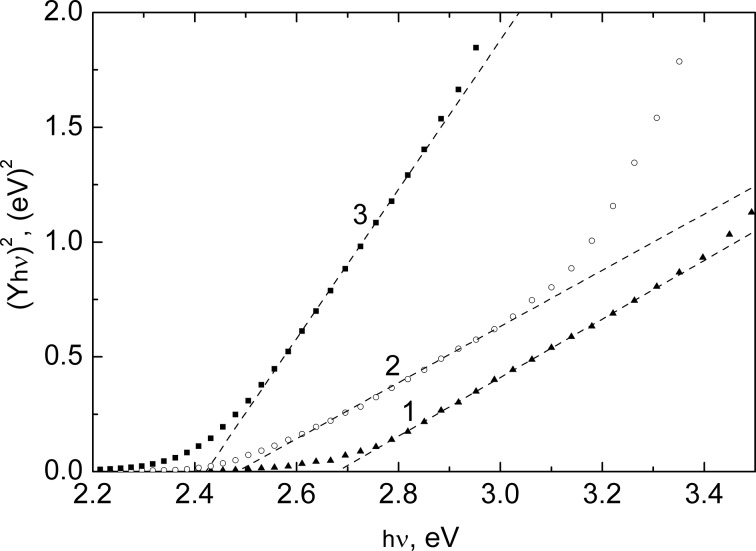
Analysis of the photocurrent spectra shown in Figure 9 in coordinates of (Y·*h*ν)^2^ versus *h*ν typical for direct optical transitions for In_2_O_3_(400)/CdS film electrodes. The number of the SILAR cycles of CdS deposition was 10 (1), 30 (2) and 50 (3).

Analysis of the photocurrent spectra in coordinates of (Y·*h*ν)^2^ versus *h*ν typical for direct optical transitions inherent in CdS shows that the bandgap of CdS decreases from 2.68 eV at 10 SILAR cycles to 2.49 eV at 30 cycles and to 2.42 eV at 50 cycles (Figure 10). The *E*_g_ value of 2.42 eV corresponds to the optical bandgap of bulk CdS. The observed increase in *E*_g_ with the decreasing number of SILAR cycles can be explained by the well-known quantum-confinement effect related to the discretization of energy levels in nanoparticles and the increase in the energy gap between LUMO and HOMO levels with the decreasing size of the semiconductor particles [25].

## Conclusion

The effect of spectral sensitization of photoelectrochemical processes on nanocrystalline mesoporous In_2_O_3_ electrodes by CdS has been revealed. This effect takes place when CdS nanoparticles are deposited on the In_2_O_3_ surface by the method of successive ion layer adsorption and reaction (SILAR) and manifests itself as a red shift of the spectral sensitivity of the electrochemical system by about 100–150 nm. The onset potential of the sensitized photocurrent for the In_2_O_3_/CdS nano-heterostructure is determined by the energy position of the oxide conduction-band edge, which is controlled by the thermal treatment of In_2_O_3_. A rise in the oxide annealing temperature from 200 to 400 °С leads to the shift of photocurrent onset potential for the In_2_O_3_/CdS nano-heterostructure by approximately 500 mV in the positive direction and results in a rise of the photocurrent by an order of magnitude.

When increasing the number of cycles of CdS deposition, gradual filling of nanopores of the indium oxide film by CdS particles occurs resulting in a rise of the optical absorbance of the In_2_O_3_/CdS films and in an increase of the photocurrent of the corresponding electrodes. Quantum-confinement effects are observed in the photocurrent spectra and lead to a bandgap increase from 2.49 to 2.68 eV when decreasing the number of SILAR cycles from 30 to 10.

The deposition of the bulk CdS on the surface of In_2_O_3_ films results in the formation of an isotype In_2_O_3_/CdS heterojunction with external narrow band-gap component. The generation and separation of photoelectrons and photoholes in this case occur primarily in the CdS at the interface with the electrolyte, and the value of the onset potential is determined by the energy position of the conduction-band edge of bulk CdS.

Raman spectroscopy data show that due to the close contact at the CdS/host-material heterojunction the lattice stress in the CdS nanoparticles rises during the deposition process, which manifests itself in changes of the position of the CdS LO phonon peak and of its width.
